# Mesenteric Arteriovenous Dysplasia/Vasculopathy: Deciphering a Rare, Deceptive, Diagnostic Dilemma

**DOI:** 10.7759/cureus.51676

**Published:** 2024-01-04

**Authors:** Sudhakar Ramamoorthy, Inuganti Venkata Renuka, Vaddatti Tejeswini, Amulya Boddapati

**Affiliations:** 1 Pathology, NRI Medical College, Chinakakani, IND

**Keywords:** mesenteric ischemia, smooth muscle collarette, fibromuscular dysplasia, mesenteric arteriovenous dysplasia/vasculopathy, vasculopathy

## Abstract

Mesenteric arteriovenous dysplasia/vasculopathy (MAVD/V) is an exceedingly rare noninflammatory vascular disorder affecting small-calibre mesenteric arteries and veins. This report details a case of a 51-year-old male diagnosed with MAVD/V following abdominal pain and vomiting. Surgical exploration revealed distinctive smooth muscle collarette around subserosal arteries and veins. The rarity of this condition, with only 13 cases reported globally, underscores the importance of recognizing this rare entity to prevent misdiagnosis. Surgical resection remains the curative approach, ensuring a disease-free state after surgery. Awareness of MAVD/V is crucial for accurate diagnosis and avoiding unnecessary prolonged management.

## Introduction

Mesenteric arteriovenous dysplasia/vasculopathy (MAVD/V) represents an uncommon, noninflammatory, and nonatherosclerotic vasculopathy predominantly affecting small-calibre mesenteric arteries and veins. Initially identified in 2016, MAVD/V remains an exceedingly rare entity, with only thirteen documented cases in the medical literature to date [1-3]. This report endeavors to present a comprehensive analysis of a 51-year-old male who, after experiencing abdominal pain, was diagnosed with this rare entity. Imaging revealed mesenteric ischemia without vessel thrombosis, leading to exploratory laparotomy and resection of a dilated jejunal segment. Microscopic examination revealed distinctive smooth muscle collarettes around subserosal arteries and veins, confirming the diagnosis. MAVD/V, typically multifocal and previously reported in the ileocecum, manifested uniquely in the jejunum in our case. Differential diagnoses, including Crohn's disease and fibromuscular dysplasia, were ruled out based on microscopic findings. Surgical resection, as demonstrated in our case and others, remains the curative treatment, ensuring a disease-free state post-surgery. Recognition of MAVD/V is crucial to prevent misdiagnosis, especially in distinguishing it from other vascular disorders, leading to optimal outcomes in this exceptionally rare condition. 

## Case presentation

A 51-year-old male presented with a one-year history of abdominal pain, weight loss, and recurrent episodes of vomiting (5-10 episodes per day). Notably, the patient denied fever and had no history of hypertension, diabetes, coronary artery disease, or cerebrovascular accident. The patient had a history of smoking and alcohol consumption, which he had given up six months ago. Physical examination revealed no palpable lymphadenopathy, splenomegaly, or hepatomegaly.

**Figure 1:**
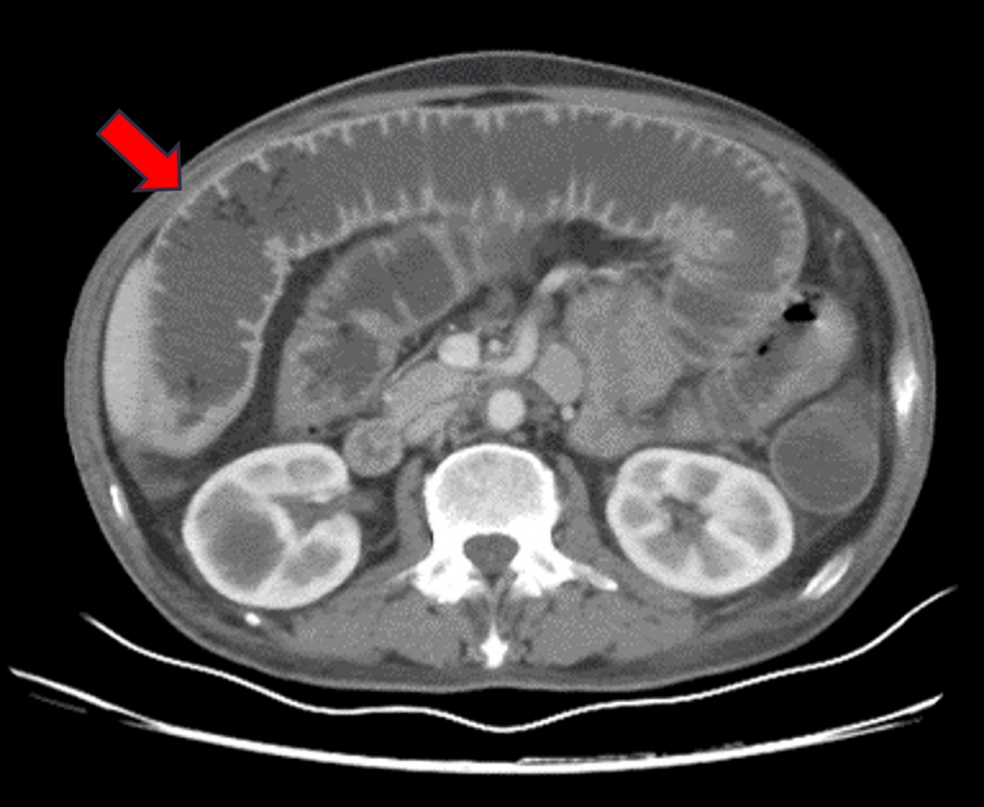
Contrast-enhanced computed tomography revealed a short segment dilatation of the jejunal loop with loss of valvulae conniventes (red arrow). No mesenteric vessel thrombus was noted.

Computed tomography imaging disclosed a short segment dilatation of the distal jejunal loop with the loss of valvulae conniventes, wall thickening, and decreased enhancement suggestive of mesenteric ischemia. Importantly, mesenteric vessel thrombosis was absent in the contrast imaging (Figure 1).

**Figure 2:**
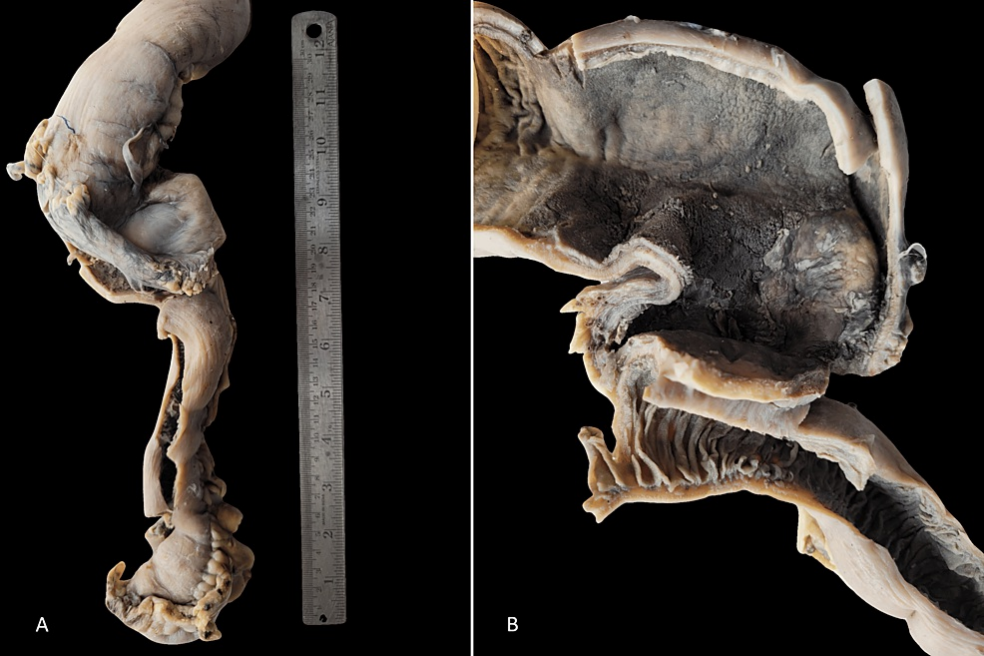
A. Gross photography displayed dilatation of the proximal jejunal segment without serosal exudates, perforation, or fat creeping. B. The mucosa exhibited loss of valvulae conniventes with a serosal adhesion between the proximal dilated segment and the distal unremarkable bowel loop.

Subsequently, exploratory laparotomy revealed distended bowel loops with a stricture approximately 50cm from the duodeno-jejunal flexure. Multiple adhesions were observed between the bowel loops. A 30cm jejunal segment was resected and side-to-side anastomosis was performed. Macroscopically, the proximal half of the specimen appeared dilated with serosal adhesions. The mucosa of the proximal dilated segment appeared flattened without signs of perforation, hemorrhagic necrosis, creeping fat, or gangrene (Figure 2). 

**Figure 3:**
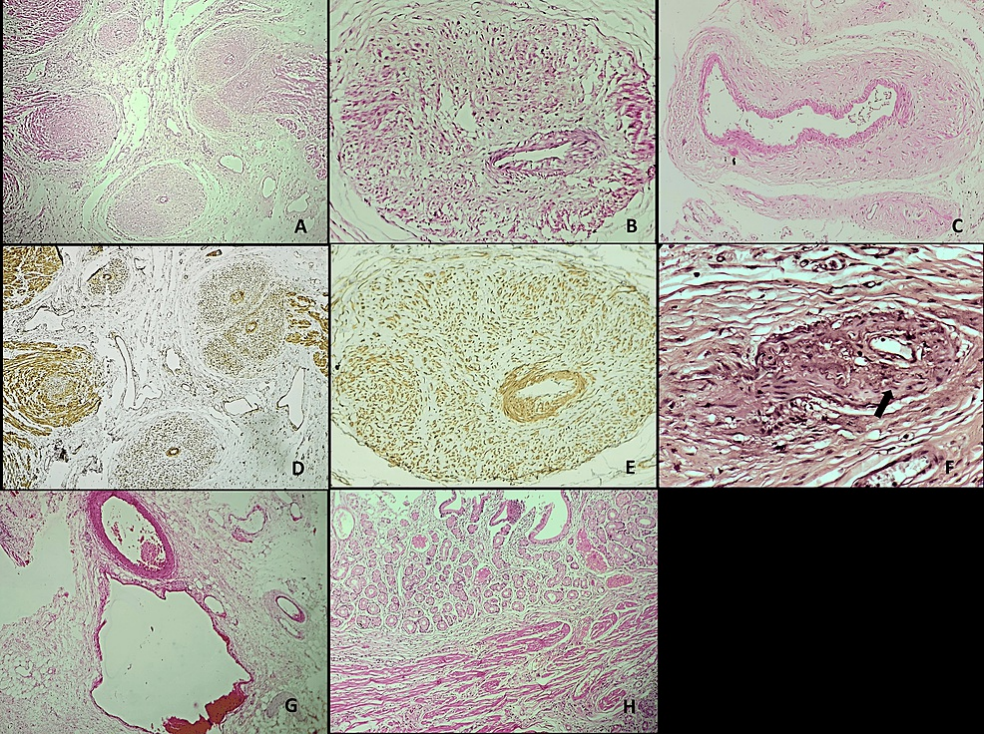
A. Low-power magnification showing a thickened wall of small-calibre subserosal arteries and veins (H&E,40x) B. High-power magnification exhibiting smooth muscle collarette around the tunica media of a subserosal artery (H&E, 200x) C. Smooth muscle collarette around the tunica media of a subserosal vein (H&E, 200x) D. Smooth muscle actin immunohistochemistry highlighting the smooth muscles around the tunica media of subserosal arteries and veins (SMA IHC 40x) E. Smooth muscle actin immunohistochemistry highlighting the smooth muscles around the tunica media of a subserosal artery (SMA IHC 200x) F. Verhoeff-van Gieson stain demonstrating disruption of the internal elastic lamina (black arrow) and intimal hyperplasia (VVG stain, 400x) G. Uninvolved medium-sized mesenteric artery and vein away from the bowel wall (H&E, 40x) H. Representative sections from the flattened mucosa show pyloric gland metaplasia with a thick muscularis mucosa. No significant inflammation, cryptitis, crypt distortion, or basal plasmacytosis was noted (H&E, 40x)

Microscopic examination demonstrated smooth muscle collarettes arranged radially around the tunica media of small-calibre arteries and veins in the subserosa (Figure 3A, 3B, 3C), as highlighted by smooth muscle actin (SMA) immunohistochemistry (Figure 3D, 3E). Van Gieson staining underscored the disruption of the internal elastic lamina of a small-calibre subserosal artery with intimal hyperplasia (Figure 3F). Medium-sized mesenteric arteries and veins located away from the bowel wall appeared unaffected (Figure 3G). The mucosa exhibited prominent pyloric gland metaplasia with thickened muscularis mucosae, while no significant inflammation, cryptitis, crypt abscess, crypt distortion, crypt loss, granuloma, or basal plasmacytosis were noted (Figure 3H). No evidence of vasculitis or malignancy was noted. Based on the presence of smooth muscle collarette involving only small-calibre subserosal arteries and veins, the absence of significant inflammation, and the lack of radiological and histopathological evidence of mesenteric vessel thrombosis, a diagnosis of MAVD/V was established. The patient exhibited no evidence of disease on her last hospital visit, which was three months after the surgery. Subsequently, the patient was lost to follow-up.

## Discussion

Mesenteric arteriovenous dysplasia/vasculopathy stands out as a distinctive, noninflammatory, and nonatherosclerotic vascular disorder primarily affecting small-calibre mesenteric arteries and veins. Since its initial recognition in 2016, a mere thirteen cases have been reported globally. The etiology remains unknown. Diagnostic criteria necessitate the presence of concentric or eccentric smooth muscle collarette around the tunica media of small-calibre subserosal artery and vein in at least two foci, accompanied by varying degrees of intimal hyperplasia or adventitial fibrosis, devoid of thrombi and significant inflammation [1-3].

Abdominal pain serves as the predominant presenting symptom, often prompting clinical suspicion towards mesenteric vessel thrombosis or Crohn’s disease. While typically manifesting as a multifocal lesion, with the ileocecum being the most frequent site, our study demonstrates the first instance of MAVD/V in the jejunum [1]. Differential diagnoses encompass Crohn’s disease, fibromuscular dysplasia (FMD), and idiopathic myointimal hyperplasia of mesenteric veins. Microscopic distinctions such as basal plasmacytosis, crypt distortion, and pericryptal granulomas effectively differentiate Crohn’s disease from MAVD/V [2]. FMD is a noninflammatory and nonatherosclerotic vasculopathy similar to MAVD/V. However, the mesenteric involvement in FMD is exceedingly rare and exclusively affects medium-sized arteries, sparing the veins. Vascular changes in FMD typically manifest as collagen deposition, as opposed to the smooth muscle collarette observed in MAVD/V [4]. Idiopathic myointimal hyperplasia predominantly involves the rectosigmoid, exclusively affecting mesenteric veins, exhibiting intimal hyperplasia with frequent thrombi in submucosal veins [5].

In contrast to other conditions, MAVD/V exclusively involves subserosal arteries and veins, characterized by the distinctive smooth muscle collarette around the tunica media. Surgical resection remains the curative treatment, with our case along with the other thirteen cases reported in the literature experiencing a disease-free state post-surgery [1-3]. Recognizing this extremely rare condition is crucial to avoid misdiagnosing it as either Crohn’s disease or FMD, thereby averting unnecessary prolonged management.

## Conclusions

In conclusion, our investigation of MAVD/V in a 51-year-old male underscores the rarity and diagnostic challenges associated with this noninflammatory vascular disorder. With only thirteen documented globally, our case contributes valuable insights, highlighting MAVD/V’s first potential manifestation in the jejunum. The distinct smooth muscle collarettes observed in microscopic analysis, coupled with the absence of inflammation and vessel thrombosis, emphasize the need for accurate diagnostic recognition. Surgical resection remains the definitive treatment, leading to a disease-free state post-surgery. Acknowledging the existence of MAVD/V is paramount to preventing misdiagnosis, particularly in distinguishing it from conditions like Crohn’s disease and fibromuscular dysplasia. Enhanced awareness is crucial for prompt and effective management, ensuring optimal outcomes in this exceptionally rare vascular disorder.
